# Supplementary material for: Bombyx batryticatus Protein-Rich Extract Induces Maturation of Dendritic Cells and Th1 Polarization: A Potential Immunological Adjuvant for Cancer Vaccine
Source: Molecules. 2021 Jan 18;26(2):476. doi: 10.3390/molecules26020476 (PMC7831066; doi:10.3390/molecules26020476)
Supplement: Supplementary file 1 [file molecules-26-00476-s001.pdf]

Supplementary materials: *Bombyx batryticatus* protein-rich extract induces maturation of dendritic cells and Th1-polarization: A potential immunological adjuvant for cancer vaccine

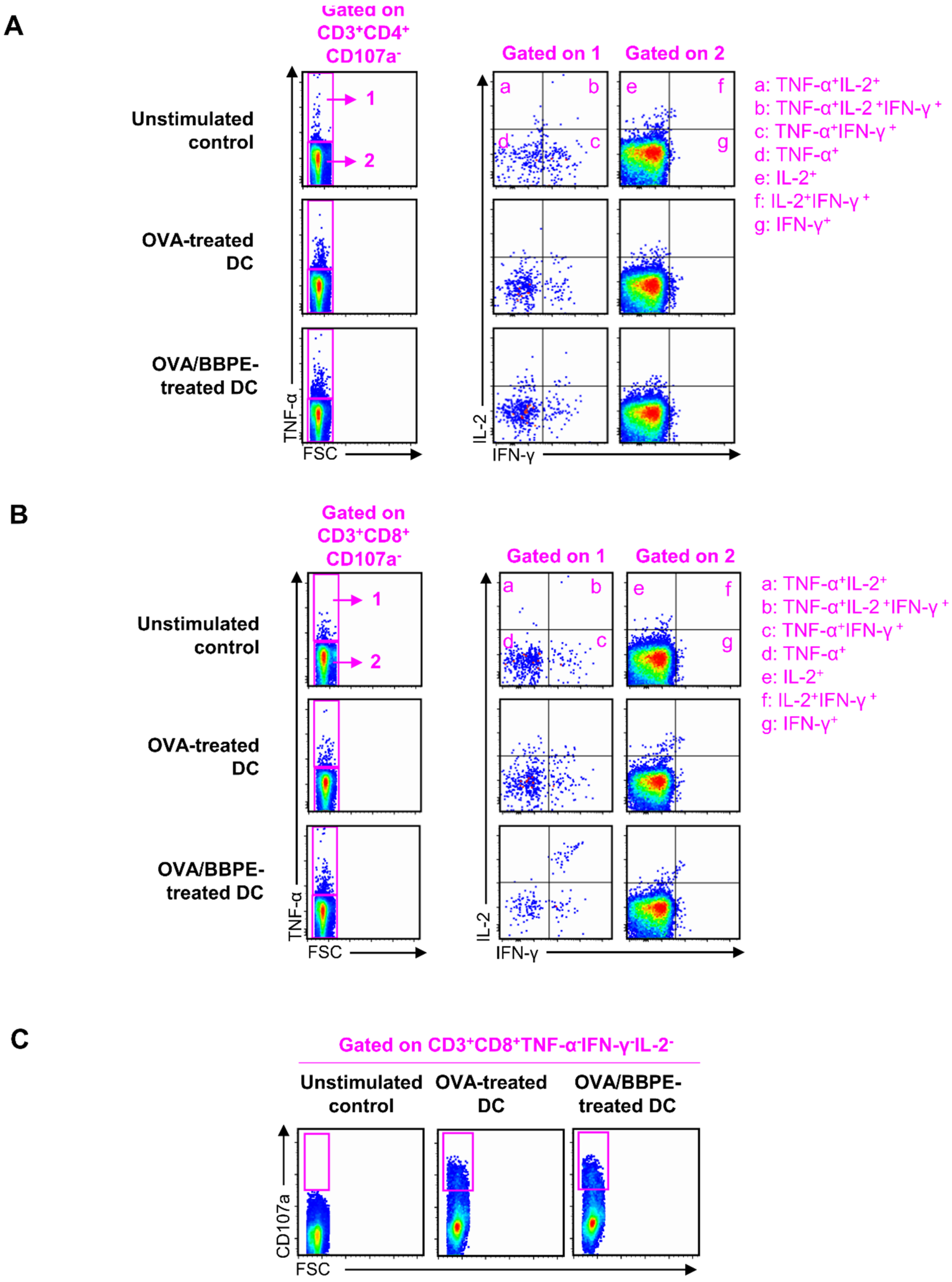

Figure S1. Example gating strategies for flow cytometry analysis for multifunctional T cells. Multifunctional-, bifunctional T cells, or each single cytokine producing T cells were gated in $CD3^+CD4^+$ (A) or $CD3^+CD8^+$ cells. CD107a (a cytotoxic granule)-producing cells were gated in $CD3^+CD8^+TNF\text{-}\alpha^-IFN\text{-}\gamma^-IL\text{-}2^-$ cells (C).

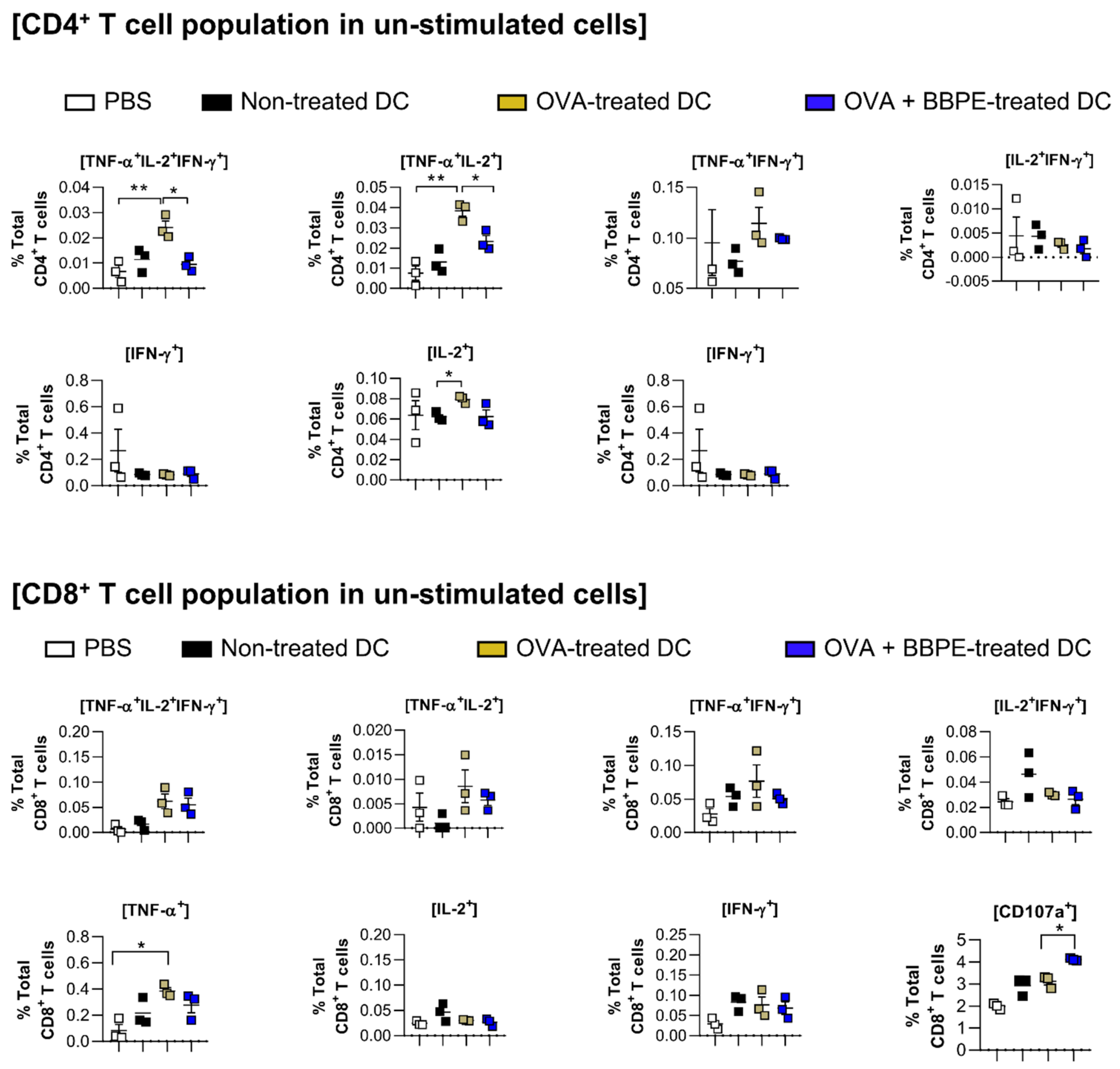

Figure S2. Percentage of multi-, bifunctional, or each single cytokine production in unstimulated cells gated on $CD3^{+}CD4^{+}$ (A) or $CD3^{+}CD8^{+}$ cells. All graphs show the mean ± SD. Statistical analysis was performed using one-way ANOVA followed by Tukey's post-hoc test. $^{*}p < 0.05$ and $^{**}p < 0.01$ represent significant differences.
